# Unraveling mate choice evolution through indirect genetic effects

**DOI:** 10.1093/evlett/qrae037

**Published:** 2024-07-22

**Authors:** Chang S Han, Diana A Robledo-Ruiz, Francisco Garcia-Gonzalez, Niels J Dingemanse, Cristina Tuni

**Affiliations:** Department of Biology, Kyung Hee University, Seoul, Korea; Department of Biology, Ludwig Maximilian University, Munich, Germany; School of Biological Sciences, Monash University, Clayton, VIC, Australia; Estación Biológica de Doñana-CSIC, Seville, Spain; Centre for Evolutionary Biology, School of Biological Sciences, University of Western Australia, Crawley, WA, Australia; Department of Biology, Ludwig Maximilian University, Munich, Germany; Department of Biology, Ludwig Maximilian University, Munich, Germany; Department of Life Science & Systems Biology, University of Turin, Torino, Italy

**Keywords:** indirect genetic effect, mate choice, genetic correlation, attractiveness

## Abstract

Attractiveness is not solely determined by a single sexual trait but rather by a combination of traits. Because the response of the chooser is based on the combination of sexual traits in the courter, variation in the chooser’s responses that are attributable to the opposite-sex courter genotypes (i.e., the indirect genetic effects [IGEs] on chooser response) can reflect genetic variation in overall attractiveness. This genetic variation can be associated with the genetic basis of other traits in both the chooser and the courter. Investigating this complex genetic architecture, including IGEs, can enhance our understanding of the evolution of mate choice. In the present study on the field cricket *Gryllus bimaculatus*, we estimated (1) genetic variation in overall attractiveness and (2) genetic correlations between overall attractiveness and other pre- and postcopulatory traits (e.g., male latency to sing, female latency to mount, male guarding intensity, male and female body mass, male mandible size, and testis size) within and between sexes. We revealed a genetic basis for attractiveness in both males and females. Furthermore, a genetic variance associated with female attractiveness was correlated with a genetic variance underlying larger male testes. Our findings imply that males that mate with attractive females can produce offspring that are successful in terms of precopulatory sexual selection (daughters who are attractive) and postcopulatory sexual selection (sons with an advantage in sperm competition), potentially leading to runaway sexual selection. Our study exemplifies how the incorporation of the IGE framework provides novel insights into the evolution of mate choice.

## Introduction

In many species with sexual reproduction, an individual’s ability to successfully mate is highly dependent on how attractive it is to opposite-sex partners (Andersson & Simmons, 2006; Rosenthal, 2017; Ryan, 2018). Individual attractiveness is typically not solely attributable to a single sexual trait but rather to a suite of sexual traits. For example, a specific combination of visual, olfactory, acoustic, and/or vibrational signals produced by an individual can determine whether it is selected as a mate by opposite-sex conspecifics (reviewed in Bro-Jørgensen, 2010; Candolin, 2003; Higham & Hebets, 2013; Moller & Pomiankowski, 1993). Thus, a single sexual trait is likely not a reliable overall indicator of individual attractiveness (Prokop & Drobniak, 2016). Focusing on single traits as measures of attractiveness thus hampers our understanding of the role of attractiveness in the evolution of mate choice.

One way to measure the overall attractiveness of a courting individual is to quantify the response of the opposite-sex chooser (Prokop & Drobniak, 2016). The chooser evaluates the combination of sexual traits expressed by the opposite-sex courter and expresses its preference through its response. For instance, in species where male copulation success depends on female consent, the duration from the initiation of male courtship to the onset of copulation (here referred to as latency to copulate) has been used to signify a male’s overall attractiveness in many studies. However, the chooser’s response does not always accurately reflect the courter’s attractiveness. For example, the latency to copulate may be more based on the female’s mating motivation than the male’s attractiveness, making it an unreliable proxy for a male’s overall attractiveness. Thus, it is necessary to assess whether the plastic response of the chooser is explained by the identity of the courter. In addition, since responses to a courter’s attractiveness can differ among choosers, averaging the responses from multiple choosers is necessary to accurately assess the attractiveness of a specific courting individual. Consequently, experimental designs should explore whether the plastic response of the chooser is influenced by the identity or genotype of the interacting courter, thereby allowing for an assessment of individual or genetic differences in the courter’s overall attractiveness. Thus, assessing individual (or genotype)-specific overall attractiveness provides a novel perspective on the evolution of mate choice compared to traditional mate choice research focusing on single sexual traits used as proxies for attractiveness.

We suggest that the “variance-partitioning approach” developed in quantitative genetics can be used to assess individual or genetic differences in overall attractiveness. It is well known that the expression of phenotypic traits can be a function of heritable traits expressed by conspecifics (Moore et al., 1997; Wolf et al., 1998, 1999). The genes of an individual determine its own phenotype, an effect that is known as “direct genetic effect” (DGE), but the genes of an individual can also influence the phenotype of another individual, an effect that is known as “indirect genetic effect” (IGE). Similarly, environmental effects specific to an individual can affect the individual’s own phenotype, referred to as a “direct environmental effect” (DEE), while the environment experienced by an individual can also affect the phenotype of another individual, referred to as an “indirect environmental effect” (IEE). Importantly, with suitable breeding designs, phenotypic variance in socially interacting traits can be partitioned into variance attributable to direct (DGEs and DEEs) versus indirect genetic and environmental effects (IGEs and IEEs). Here, IGEs and IEEs on the response of the chooser reflect the variance in the response of the chooser that is attributable to the combined effects of all attractive traits of the opposite-sex courter (e.g., Edward et al., 2014; Hall et al., 2013; Han et al., 2020; Petfield et al., 2005). Choosers may evaluate many sexual traits of the opposite-sex courter during mate choice, but only some (or combinations) of traits will be genetically variable and produce IGEs on the response of the chooser. Thus, an advantageous aspect of employing variance-partitioning approaches is the ability to estimate individual or genetic variation in overall attractiveness in the absence of information on the number and type of traits that underpin attractiveness (McGlothlin & Brodie, 2009).

The assessment of individual overall attractiveness using the variance-partitioning approach can also enable the test of hypotheses related to the evolution of mate choice and attractive traits. Significant IGEs on the response of the chooser indicate heritable overall attractiveness of the opposite-sex courter. Moreover, the presence of heritable attractiveness suggests that individuals may gain indirect genetic benefits by choosing mates with attractive traits (Prokop et al., 2012), thereby influencing the evolution of mate choice. If the overall attractiveness of the courter is indeed heritable, an opposite-sex chooser mated with an attractive courter can expect to produce attractive offspring. For instance, in the field cricket *Gryllus bimaculatus*, significant and positive IGEs were observed for male postcopulatory guarding activity and female latency to mate, indicating that the attractiveness of both sexes is heritable (Han et al., 2020). This implies that both males and females can potentially gain indirect genetic benefits by choosing attractive mates because mating with attractive mates can lead to the production of attractive offspring. These genetic benefits in turn can explain the evolution of mutual mate choice in *G. bimaculatus*. Therefore, assessing the genetic variance in a courter’s overall attractiveness through the calculation of IGEs on the chooser’s responses can provide insights into the evolution of mate choice.

**Figure 1.**
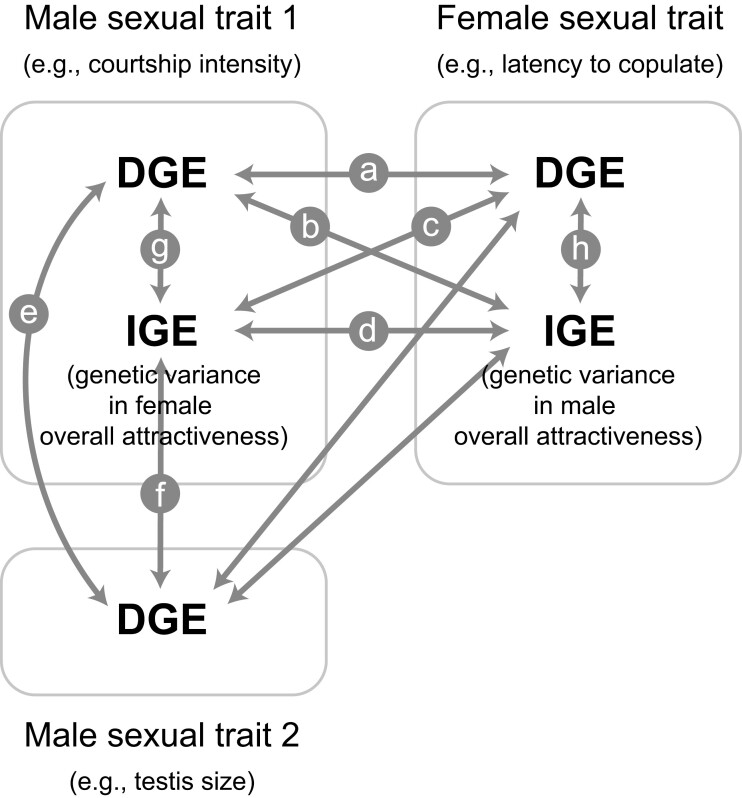
Complex within-sex and cross-sex genetic correlation structures considering both direct (DGEs) and indirect genetic effects (IGEs) on male and female sexual traits. The hypothetical scenarios examined are as follows: (a) DGE–DGE, (b, c) DGE–IGE, and (d) IGE–IGE cross-sex correlations, and (e) DGE–DGE and (f, g, h) DGE–IGE within-sex correlations. Specifically, cross-sex DGE–DGE associations indicate that (a) a genetic variance associated with male courtship is correlated with a genetic variance related to female motivation to copulate. Within-sex DGE–DGE associations show that (e) a genetic variance related to male courtship intensity is correlated with a genetic variance related to male testis size. In addition, within-sex (within-male) DGE–IGE associations show that a genetic variance related to female attractiveness that elicits intense male courtship is correlated with (g) a genetic variance related to male courtship intensity or (f) a genetic variance related to male testis size. Similarly, within-sex (within-female) DGE–IGE associations indicate that a genetic variance influencing male attractiveness is correlated with a genetic variance related to female motivation to copulate. Cross-sex DGE–IGE associations reveal that (b) a genetic variance related to male courtship is correlated with male attractiveness or that (c) a genetic variance related to female motivation to copulate is associated with a genetic variance influencing female attractiveness. Finally, cross-sex IGE–IGE associations show that (d) there are genetic correlations between male and female attractiveness.

Moreover, applying multivariate versions of variance-partitioning approaches facilitates the study of intricate genetic structures associated with overall attractiveness, enabling further investigation into which traits are genetically linked to the evolution of mate choice (Figure 1). Notably, IGEs on labile mating behavior in one sex (representing the genetic basis of the opposite sex’s attractiveness) can be correlated with DGEs on other traits within the same or the opposite sex (i.e., within-sex or cross-sex DGE–IGE correlations; b, c, f, g, and h in Figure 1). For example, positive associations between DGEs on a specific courter trait (e.g., courtship intensity) and IGEs on chooser responses (additive genetic variance in the courter’s overall attractiveness) suggest that the courter trait serves as an indicator of the overall attractiveness of the courter at the genetic level (cross-sex DGE–IGE correlations; b or c in Figure 1). Petfield et al. (2005) showed that the expression of cuticular hydrocarbons (CHCs) in *Drosophila serrata* males was influenced by female genotype (i.e., an IGE on male CHCs), indicating additive genetic variation in female attractiveness. Moreover, female overall attractiveness was genetically correlated with female body mass, suggesting that male mate choice was influenced by female body mass (Petfield et al., 2005). Furthermore, courter attractiveness (as measured by IGEs on the chooser response) may be positively associated with DGEs on the chooser’s fitness-related traits (within-sex DGE–IGE correlations; f, g, and h in Figure 1). This indicates that choosers exercising mate choice reap indirect benefits by producing both attractive and viable offspring (e.g., sexy sons and viable daughters), highlighting the importance of genetic benefits in mate choice. Thus, the application of the IGE framework and multivariate perspective in the study of attractiveness holds substantial promise for advancing our understanding of the evolution of mate choice.

Here, we estimated DGEs- and IGEs-associated within- and cross-sex genetic architectures of multiple pre- and postcopulatory sexual traits in the field cricket *G. bimaculatus*, and tested the existence of possible genetic benefits in the evolution of mate choice. *Gryllus bimaculatus* males fight aggressively to control breeding territories, from which they sing to attract partners (Adamo & Hoy, 1995; Simmons, 1986b). Once females are in close proximity, males also perform close-range courtship songs (Simmons, 1986a). Females then actively mount preferred males and are inseminated through discharge of the male’s spermatophore. Females are polyandrous (Tregenza & Wedell, 1998), which leads to postcopulatory sperm competition and selection (Bretman & Tregenza, 2005; Simmons, 1987). Both sexes are known to exert mate choice (Han et al., 2020), and the evolution of mate choice in *G. bimaculatus* females is suggested to be due to genetic compatibility rather than good genes (Rodríguez-Muñoz et al., 2008; Tregenza & Wedell, 1998). In our study, males from a pedigreed population (with a paternal half-sib breeding design) were consecutively paired with up to three females (i.e., sequential choice tests; Dougherty & Shuker, 2015), allowing us to repeatedly score mating behaviors such as male latency to sing, female latency to mount, and male postcopulatory mate-guarding intensity (a postcopulatory sexual trait performed to prevent mated females from removing the male’s spermatophore); we could then determine how individuals vary in their mating behavior toward different opposite-sex partners. We also measured morphological traits, including male and female body mass, male mandible size (a precopulatory sexual trait used during intrasexual contests), and testis size (a male postcopulatory sexual trait used as a proxy for sperm production). We then used a multivariate animal model to assess genetic (co)variance structures among all DGEs and IGEs on a suite of sexual traits expressed in males and/or females. This enabled us to test (1) whether the overall attractiveness of *G. bimaculatus* (as measured by IGEs on the chooser response) was heritable in both males and females, (2) which morphological or behavioral traits genetically contributed to the overall attractiveness within a sex, and (3) how overall attractiveness was genetically associated with traits of the opposite sex. Finally, we explored how assessing the genetic architecture of overall attractiveness contributes to improve our understanding of the evolution of mate choice.

## Materials and methods

### Animal rearing and pedigreed population

The animals used in this study were derived from a parental population of wild crickets collected in Tuscany, Italy, during the summer of 2014 and maintained in a climate-controlled room at the Ludwig Maximilian University of Munich (Germany), under a constant temperature (26 °C) and humidity (65%) with a 14:10 hr light:dark cycle (see Supplementary Text S1 for details on the maintenance of the stock population). Once nymphs in the stock population reached the last instar, they were isolated in individual plastic containers (10 × 10 × 9 cm^3^) provided with shelter, food, and water and raised to adulthood. Sexually mature virgin adult crickets were assigned to a paternal half-sib breeding design (Lynch & Walsh, 1998). Specifically, we randomly selected 35 unrelated males (sires) from the laboratory population and allowed each male to fertilize the clutches of two unrelated virgin females (dams) consecutively. Because of some breeding failures, the final design yielded a total of 66 full-sib families (32 pairs of paternal half-sib families and 2 full-sib families without paternal half-sibs). Please see Supplementary Text S2 for details on the collection of experimental individuals from the population subjected to breeding design.

### Mating trials and measurements of behavioral and morphological traits

Animals were tested 2 weeks after final eclosion to maturity. Mating trials took place over 3 consecutive days, in which each male was randomly paired with 1–3 unmated females, one female per day. A total of 826 mating assays were performed, involving 310 males and 747 females. Before each mating trial, the body mass of individuals of both sexes was measured to the nearest 0.01 g using a digital scale (Kern PKT, Kern & Sohn GmbH, Germany). A 2 × 2 mm square of colored tape (male = red, female = blue) was attached to the crickets’ pronotum to identify cricket sex.

All details of our assay procedures have been described elsewhere (Han et al., 2020; Han & Dingemanse, 2017). Briefly, mating trials were performed in a mating arena (16 × 16 × 20 cm) equipped with a high-resolution video camera (Basler GenlCam, Germany). A randomly selected male and female were placed in the arena and allowed to interact for 23 min. Once the mating trials were completed, the crickets were placed in individual vials at −20 °C for further morphological measurements. Later, we thawed the crickets to measure the mandible size and testis size (see Supplementary Text S3 for details).

From the video recordings, two behavioral traits were scored to the millisecond using JWatcher software (version 0.9) (Blumstein & Daniel, 2007): (1) male latency to sing, defined as the time interval from when males and females came into physical contact (i.e., antennation) to the start of male singing (here, used as an indicator of male mating motivation and courtship), and (2) female latency to mount, defined as the time interval from male courtship initiation (i.e., the start of male song) to the female’s first mounting attempt, regardless of whether successful spermatophore transfer occurred. In addition, (3) the intensity of postcopulatory mate guarding was scored using EthoVision software (Noldus EthoVision XT 10, Noldus Information Technology) and measured as the average distance between males and females (in cm) after copulation until the end of the trial.

### Statistical analyses

First, we separately assessed sources of variation in each trait using univariate mixed-effects animal models. Then we assessed within- and cross-sex genetic correlations using bivariate mixed-effects animal models. All models were implemented using ASReml (v. 4.1, VSN Interaction, Hemel Hempstead, UK) and solved using restricted maximum likelihood. We checked model assumptions concerning normality of residuals by visual inspection of residual plots. All trait scores were *z*-transformed prior to variance partitioning to facilitate comparisons among traits.

#### Univariate analyses

In the univariate mixed-effects models, the focal behavioral trait was fitted as the response variable and testing order (covariate: first, second, or third mating by the focal male) and testing shelf (two-level factor: lower vs. upper) were fitted as fixed effects. Using the pedigree information, we also partitioned phenotypic variance into genetic (DGEs, IGEs) and nongenetic components (DEEs, IEEs). Because male (latency to sing and guarding intensity) and female (latency to mount) mating behaviors were repeatedly assessed over multiple male–female interactions with different opposite-sex partners, we partitioned the variance attributable to male and female identity into genetic (additive genetic effects of males: *V*_Am_; additive genetic effects of females: *V*_Af_) and nongenetic (permanent environmental effect of males: *V*_PEm_; permanent environmental effect of females: *V*_PEf_) components. In addition, we included the covariance (COV_Am,Af_) between *V*_Am_ and *V*_Af_, although the values were sometimes bounded because of a nonsignificant *V*_Am_ or *V*_Af_. Based on those (co)variance components, we estimated the total heritable variance using the equation (*V*_TBV_ = *V*_Am_ + *V*_Af_ + 2COV_Am,Af_) which specifically holds for a dyadic interaction (group number (*n*) = 2; equation (6) in Bijma et al., 2007). We presented results related to the total heritable variances and their implications in the Supplementary Text S4.

In the univariate mixed-effects models, we excluded the variance attributable to the identity of the opposite-sex partner when fitting the morphological trait as the response variable. This was because the expression of morphological traits (e.g., body mass, mandible size, or testis size) is independent of traits expressed by opposite-sex partners. Additionally, in the univariate models where mandible size or testis size was fitted as the response variable, we could not consider permanent environmental effects because these traits were measured only once. In contrast, in the univariate model where body mass was fitted as the response variable, we partitioned the among-individual variance in body mass into genetic and nongenetic (permanent environmental) components because male body mass was repeatedly measured.

#### Multivariate analyses

Next, we fitted bivariate mixed-effects animal models to estimate within- or cross-sex genetic correlations. We constructed a model fitting two traits within a sex or two traits, either homologous or heterogenous across sexes, that both showed significant additive genetic effects (i.e., significant *V*_Am_ or *V*_Af_, respectively) as the two response variables. In the multivariate analysis, the latency scores were multiplied by −1 such that higher scores reflected greater motivation to mate or greater attractiveness, for ease of interpretation of correlations related to DGEs or IGEs on the latency. In the model, we also partitioned the variance attributable to male/female identity into genetic and nongenetic components, allowing us to determine the additive genetic variation–covariation matrix and genetic correlation structures. In the model, permanent environmental variances in the sizes of testes and mandibles were constrained to zero because each male’s testis size and mandible size were measured only once. The permanent environmental covariation between male and female reproductive traits was also fixed at zero. We did not include fixed factors to facilitate convergence. As there were no significant additive genetic effects from males or females on male guarding intensity, we were unable to estimate genetic correlations between male guarding intensity and the other traits.

#### Significance test

The significance of fixed effects was determined using Wald *F* tests, and the significance of variances was determined using likelihood ratio tests (LRTs), calculated as twice the difference in the log likelihood between models where the focal random effect (variance) was included or removed. The *p* value was calculated using a mixture of *p*(χ^2^, df = 0) and *p*(χ^2^, df = 1), denoted “χ^2^_0/1_” below (Self & Liang, 1987; Visscher, 2006). When we assessed the significance of genetic correlations in multivariate models, we calculated the LRT as the difference in deviance between the full model and a model where the focal correlation was constrained to zero, assuming one degree of freedom (*p*(χ^2^, df = 1)). In addition, although our analysis includes the same data in many different tests (univariate and bivariate), we did not perform multiple testing corrections in our analysis. This was because multiple testing corrections could reduce Type I errors but substantially increase the Type II error rate.

## Results

### Univariate analyses

**Figure 2.**
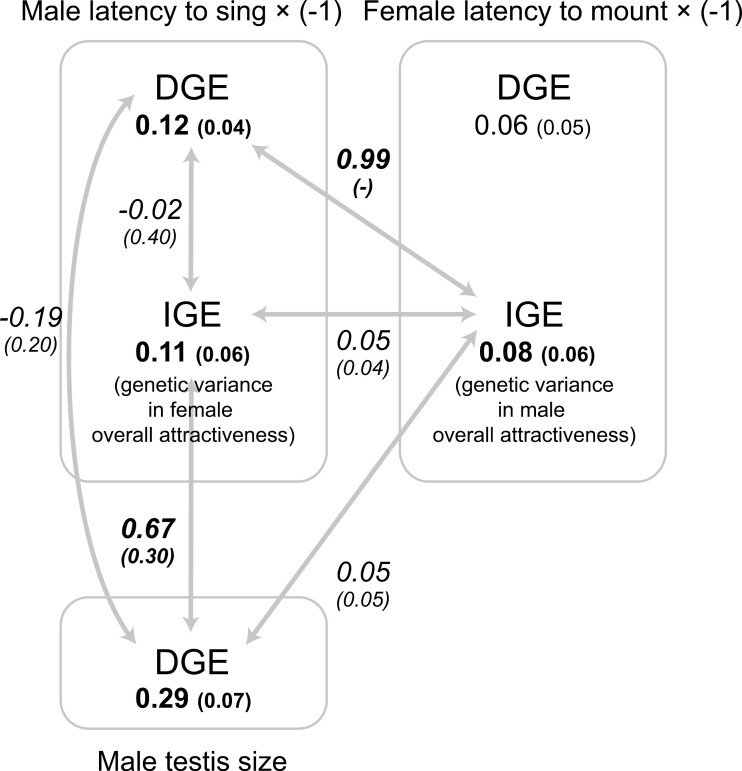
Within-sex and cross-sex genetic correlation structures including direct (DGEs) and indirect genetic effects (IGEs). The latency scores were multiplied by −1 prior to analysis. Variances (DGEs or IGEs) and correlation coefficients (the numbers on arrows) are provided with standard errors in parentheses. Significant variances and correlations (*p* < 0.05) are indicated in bold.

DGEs and IGEs both explained significant variation in male latency to sing (Figure 2; Supplementary Table S1). That is, male genotypes differed in their latency to sing (a DGE), and male latency to sing also depended on the female genotype (an IGE). This finding of significant IGEs on male latency to sing indicates that male latency to sing reflects female attractiveness to some extent. However, DGEs and IGEs on male latency to sing (reverse-scored) were not associated (Figure 2; Table 1 and Supplementary Table S2).

**Table 1.** Within-sex and cross-sex genetic correlations for multiple pre- and postcopulatory traits. Latency values were reverse-scored, as indicated by an asterisk (*).

Within-sex genetic correlations	*r* (*SE*)	Test of *r* = 0
Male latency to sing * (DGE) − Male body mass (DGE)	0.01 (0.16)	χ^2^_1_ = 0.00, *p* = 0.50
Male latency to sing * (DGE) − Male testis size (DGE)	−0.19 (0.20)	χ^2^_1_ = 0.92, *p* = 0.34
Male latency to sing * (DGE) − Male mandible size (DGE)	0.06 (0.19)	χ^2^_1_ = 0.10, *p* = 0.75
Male body mass (DGE) − Male testis size (DGE)	**0.44 (0.10)**	**χ** ^**2**^_**1**_ **= 17.26, *p* < 0.001**
Male body mass (DGE) − Male mandible size (DGE)	**0.95 (0.03)**	**χ** ^**2**^_**1**_ **= 230.84, *p* < 0.001**
Male testis size (DGE) − Male mandible size (DGE)	**0.47 (0.11)**	**χ** ^**2**^_**1**_ **= 16.00, *p* < 0.001**
Male latency to sing * (IGE)^a^ − Male latency to sing * (DGE)	−0.02 (0.40)	χ^2^_1_ = 0.00, *p* = 0.50
Male latency to sing * (IGE)^a^ − Male body mass (DGE)	0.11 (0.31)	χ^2^_1_ = 0.12, *p* = 0.73
Male latency to sing * (IGE)^a^ − Male testis size (DGE)	**0.67 (0.30)**	**χ** ^**2**^_**1**_ **= 4.33, *p* = 0.04**
Male latency to sing * (IGE)^a^ − Male mandible size (DGE)	0.26 (0.33)	χ^2^_1_ = 0.64, *p* = 0.42
Female body mass (DGE) − Female latency to mount * (IGE)^b^	−0.05 (0.30)	χ^2^_1_ = 0.02, *p* = 0.89

Cross-sex genetic correlations	*r* (*SE*)	Test of *r* = 0
Female latency to mount * (IGE)^b^ − Male body mass (DGE)	0.11 (0.19)	χ^2^_1_ = 0.32, *p* = 0.57
Female latency to mount * (IGE)^b^ − Male testis size (DGE)	−0.27 (0.23)	χ^2^_1_ = 1.36, *p* = 0.24
Female latency to mount * (IGE)^b^ − Male mandible size (DGE)	0.15 (0.22)	χ^2^_1_ = 0.42, *p* = 0.52
Male latency to sing * (DGE) − Female latency to mount * (IGE)	**0.99 (–)**	**χ** ^**2**^_**1**_ **= 20.16, *p* < 0.001**
Male latency to sing * (DGE) − Female body mass (DGE)	0.05 (0.26)	χ^2^_1_ = 0.04, *p* = 0.84
Male latency to sing * (IGE)^a^ − Female body mass (DGE)	−0.09 (0.20)	χ^2^_1_ = 0.20, *p* = 0.65
Male latency to sing * (IGE)^a^ − Female latency to mount * (IGE)^b^	0.05 (0.04)	χ^2^_1_ = 1.32, *p* = 0.25
Female body mass (DGE) − Male body mass (DGE)	**0.53 (0.16)**	**χ** ^**2**^_**1**_ **= 8.12, *p* = 0.004**
Female body mass (DGE) − Male testis size (DGE)	0.21 (0.20)	χ^2^_1_ = 1.00, *p* = 0.32
Female body mass (DGE) − Male mandible size (DGE)	**0.56 (0.18)**	**χ** ^**2**^_**1**_ **= 6.80, *p* = 0.009**

*Note.* DGE = direct genetic effect; IGE = indirect genetic effect.

Significant variances and correlations (*p* < 0.05) are indicated in bold.

^a^Indicates (1) genetic variation in female attractiveness and (2) the existence of male preference for specific female genotypes.

^b^Indicates (1) genetic variation in male attractiveness and (2) the existence of female preference for specific male genotypes.

In addition, the variation in female latency to mount was explained by IGEs but not by DGEs (Figure 2; Supplementary Table S1). Significant IGEs on female latency to mount implies that female latency to mount reflects male attractiveness. Despite positive additive genetic components in precopulatory behavioral traits, additive genetic components of male guarding activity were not different from zero (Supplementary Table S1).

In addition to behavioral traits, male and female pre- and postcopulatory morphological traits (male and female body size, male mandible size, and male testis size) were highly heritable (i.e., exhibited strong DGEs, Figure 2; Supplementary Table S1).

### Multivariate analyses

#### Within-sex DGE–DGE correlations

We found significant positive DGE–DGE correlations among male morphological traits (Table 1 and Supplementary Table S2), indicating that the heavier male genotypes had larger mandibles and larger testes. However, there were no significant within-sex DGE–DGE correlations between morphological and behavioral traits in either sex (Table 1 and Supplementary Table S2).

#### Cross-sex DGE–DGE correlations

DGEs on female body mass were positively correlated with DGEs on male body mass and mandible size but not with DGEs on male testis size (Table 1 and Supplementary Table S2). DGEs on male latency to sing were not associated with DGEs on female body mass (Table 1 and Supplementary Table S2).

#### Within-sex (cross-trait) DGE–IGE correlations

IGEs on male latency to sing (reverse-scored; reflecting female attractiveness) were positively correlated with DGEs on male testis size (*r*(*SE*) = 0.67 (0.30), Figure 2; Table 1 and Supplementary Table S2) but not with DGEs on male body mass or mandible size (Table 1 and Supplementary Table S2). That is, a genetic variance related to larger male testes was associated with a genetic variance related to female attractiveness. IGEs on female latency to mount (reverse-scored; reflecting male attractiveness) were not related to DGEs on female body mass (Figure 2; Table 1 and Supplementary Table S2).

#### Cross-sex DGE–IGE correlations

We found a positive correlation between DGEs on male latency to sing (reverse-scored) and IGEs on female latency to mount (reverse-scored; reflecting male attractiveness) (Figure 2; Table 1 and Supplementary Table S2). That is, male genotypes that rapidly initiated courtship song were mounted more rapidly by females (*r* = 0.99, Figure 2; Table 1 and Supplementary Table S2). However, IGEs on female latency to mount (reverse-scored; reflecting male attractiveness) were not associated with DGEs on male morphological sexual traits (Table 1 and Supplementary Table S2). IGEs on male latency to sing (reverse-scored; reflecting female attractiveness) were not related to DGEs on female body mass (Table 1 and Supplementary Table S2).

#### Cross-sex IGE–IGE correlations

IGEs on male latency to sing (reverse-scored; reflecting female attractiveness) were not associated with IGEs on female latency to mount (reverse-scored; reflecting male attractiveness). That is, a genetic variance related to female attractiveness was independent of the genetic variance related to male attractiveness (Figure 2; Table 1 and Supplementary Table S2).

## Discussion

Previous studies on within- or cross-sex genetic correlation structures have predominantly examined DGEs on traits, often neglecting IGEs (Poissant et al., 2010). Moreover, studies of DGE–IGE correlations have been limited to within-sex analyses (Brinker et al., 2015; Fisher et al., 2019; Han et al., 2018; Moorad & Nussey, 2016; Peeters et al., 2012; Santostefano et al., 2017; Sartori & Mantovani, 2013; Thomson et al., 2017; Wilson et al., 2009). However, our study, using multivariate versions of variance-partitioning approaches, demonstrated that IGEs can be associated with both DGEs and IGEs on other traits not just within a sex but also across sexes (Figure 2).

First, within-sex cross-trait DGE–IGE correlations underscore the genetic benefits in the evolution of mate choice and attractive traits. We found a positive genetic correlation between female attractiveness (measured by IGEs on male mating behavior) and male testis size in *G. bimaculatus*. This correlation suggests that mate choice can produce offspring successful at both pre- and postcopulatory stages, supporting the genetic benefits of mate choice in *G. bimaculatus*. In addition, cross-sex cross-trait DGE–IGE correlations can identify traits contributing to overall attractiveness. We found a positive correlation between IGEs on female mating behavior and DGEs on male latency to sing (reverse-scored) in *G. bimaculatus*, indicating that more attractive males exhibited a shorter latency to sing toward females. Therefore, we highlight that these complex DGE–IGE correlations offer valuable insights into the evolutionary dynamics of mate choice (Figure 1).

### Applications of IGEs in identifying specific sexual traits associated with mate attraction

The estimation of cross-sex cross-trait DGE–IGE correlations represents an extremely useful approach for identifying which traits contribute to overall attractiveness at the genetic level (correlations b and c in Figure 1). In many animal taxa, overall attractiveness is influenced in a non-additive manner by multiple components within the same sensory modality or across modalities (Candolin, 2003; Hebets & Papaj, 2005; Partan & Marler, 2005). Previous studies have investigated the relationship between individual traits and mating success using multivariate selection analyses, attempting to elucidate the role of each trait in mating success (Lande & Arnold, 1983). Individual mating success can reflect overall attractiveness, but it is not a perfect index of attractiveness because mating success is also determined by ecological and social factors, including dominance (e.g., the outcome of fights), as well as individual attractiveness, which incorporates all phenotypic components of precopulatory sexual selection. This study introduces the combination of multivariate analyses and the IGE framework to estimate cross-sex cross-trait DGE–IGE correlations to identify specific traits affecting mate attraction. Here, genetic variance in overall attractiveness of the courter can be estimated by IGEs on the response of the opposite-sex chooser. Moreover, attractive traits can be assessed by the association between the courter’s overall attractiveness (IGEs or IGEs + IEEs on the response of the chooser) and the specific traits of the courter (correlations b and c in Figure 1). To the best of our knowledge, only one study to date has evaluated the “genetic-level” contribution of individual attractive traits to overall attractiveness using this approach. In *D. serrata*, there was a positive association between IGEs on the expression of male CHCs (genetic variation in female attractiveness) and the genetic variation in female body mass, suggesting that female body mass is an attractive trait (Petfield et al., 2005). In addition, our study in *G. bimaculatus* has found a positive genetic correlation between male overall attractiveness (measured by IGEs on female latency to mount) and male mating motivation (Figure 2), suggesting that females readily accept reproductively motivated male genotypes that promptly initiate courtship after recognizing females.

In addition to the estimation of cross-sex cross-trait DGE–IGE correlations, specific attractive traits can be detected using “trait-based approaches” modeling IGEs (McGlothlin & Brodie, 2009). To represent IGEs, the trait-based approach estimates social responsiveness, Ψ, as a regression coefficient by regressing the phenotype of a focal individual or genotype on the phenotype of an interacting partner (Moore et al., 1997). In the mating context, the trait-based approach calculates Ψ based on the effects of specific phenotypic traits of the courting sex (e.g., males) on the response (e.g., mate choice) of the chooser sex (e.g., females) (e.g., Bailey & Hoskins, 2014; Bailey & Zuk, 2012; Chenoweth et al., 2010; Signor et al., 2017). Thus, like the variance-partitioning approach, this approach also assesses IGEs, by calculating Ψ, and the estimation of Ψ allows the identification of specific attractive traits. In addition, the genetic-level Ψ matrix can be estimated using the product of the IGE-DGE covariance matrix and the inverse of the DGE matrix, which are variance components calculated via the variance-partitioning approach (McGlothlin & Brodie, 2009). Hence, we propose that cross-sex cross-trait DGE–IGE correlations as well as genetic-level Ψs should be used to evaluate the relative contribution of each attractive trait to the overall attractiveness of the courter at the genetic level.

### Applications in IGEs to the study of mate choice evolution

Our finding of heritable variation in overall attractiveness implies that mate choice can confer indirect genetic benefits, as individuals that select attractive mates have the potential to produce offspring that are highly attractive (Prokop et al., 2012). Notably, in our study, the positive genetic correlation between female attractiveness and male testis size suggests that male crickets that mate with attractive females can produce both attractive (at the precopulatory stage) daughters but also competitive (at the postcopulatory stage) sons (Figure 2). Furthermore, when overall attractiveness of the courter is heritable, and when the opposite-sex chooser exerts mate choice (i.e., choosers prefer attractive courters), Fisherian runaway selection is likely to occur (Fisher, 1958; Henshaw & Jones, 2020; Mead & Arnold, 2004). For the evolution of mate choice through Fisherian runaway sexual selection (Fisher, 1915, 1930, 1958) or through Lande–Kirkpatrick models (Kirkpatrick, 1982; Lande, 1981), sons must inherit genes related to both their father’s attractiveness and their mother’s preference for this attractiveness. While we did not explicitly calculate the genetic correlation between attractive traits and the preference for those traits, heritable attractiveness of the courter (i.e., IGEs on the mating response of the chooser) can form the basis for Fisherian runaway sexual selection (Fisher, 1915, 1930). The chooser’s preference for heritable attractiveness of the courter and nonrandom mating can lead to linkage disequilibrium between attractiveness and the preference for attractiveness (Lande, 1981). This, in turn, can contribute to a self-reinforcing coevolutionary process between male overall attractiveness and female preference for overall attractiveness (Fisher, 1958; Henshaw & Jones, 2020; Mead & Arnold, 2004).

The self-reinforcing process leading to Fisherian runaway sexual selection may not operate if individual attractiveness does not enhance mating success that would constitute a Fisherian indirect benefit. However, given that male attractiveness measured by female latency to mount was highly associated with male mating success in the field cricket *Teleogryllus commodus* (Shackleton et al., 2005), our metrics of attractiveness (male latency to sing and female latency to mount) may similarly be closely linked to individual mating success in the field cricket *G. bimaculatus*. This relationship could contribute to the self-reinforcing process between a courter’s overall attractiveness and a chooser’s preference for that attractiveness. Therefore, the heritable overall attractiveness observed in both sexes of *G. bimaculatus* suggests that indirect genetic benefits provide the conditions necessary for the evolution of mate choice in this species, leading to the development of an attractiveness-preference correlation and runaway selection.

Thus, our application of the IGE framework has generated evidence for heritable attractiveness among courters and the presence of mate preference in opposite-sex choosers (i.e., sexual selection of the courter’s traits related to attractiveness), which can be used to infer a genetic basis for Fisherian runaway sexual selection. While accumulating empirical evidence supports runaway selection (Prokop et al., 2012; Prokuda & Roff, 2014; Rodríguez, 2020), the genetic correlation between a signal trait and preference for that trait is frequently weak (Greenfield et al., 2014). This might be due to the small sample size used in the calculation of cross-sex genetic correlations (Sharma et al., 2016) and the low number of partners sampled when researchers determine preference (Roff & Fairbairn, 2014). Another possible reason is that mate preference depends on the contribution of multiple sexual traits to the overall attractiveness of opposite-sex partners. Preferences for overall attractiveness, shaped by various sexual traits in the courter, may differ from preferences for specific sexual traits (Prokop & Drobniak, 2016). As a result, even if there is a positive genetic correlation between overall attractiveness and preference for overall attractiveness, the genetic correlation between a single sexual trait and preference for that trait might not be positive. However, significant IGEs on the chooser response indicate that there is genetic variation in the overall attractiveness of the opposite-sex courter as well as potential indirect genetic benefits that the chooser can obtain from mating with attractive opposite-sex courters, laying the groundwork for runaway selection (Fisher, 1958; Henshaw & Jones, 2020; Mead & Arnold, 2004). Therefore, considering IGEs is critical when testing for runaway selection.

The quantification of (within- or cross-sex) cross-trait DGE–IGE correlations also represents a useful approach for testing the good genes hypothesis regarding the evolution of mate choice. Traditionally, the good genes hypothesis has been examined by assessing the association between the courter’s attractive trait (e.g., signal intensity) and the courter’s offspring viability (e.g., parasite resistance) (Achorn & Rosenthal, 2020). However, this approach may not capture the true relationship between courter attractiveness and offspring viability if a specific signal trait does not reflect the overall attractiveness of the courting individuals. Estimating IGEs on mating behavior overcomes this limitation. Within the IGE framework, a positive correlation between IGEs on chooser mating behavior (representing the genetic basis of the courter’s attractiveness) and DGEs on the viability of the courter’s offspring would support the role of the good genes hypothesis in the evolution of mate choice (Figure 1). For example, if there is a positive genetic correlation between male immunity and attractiveness as measured by the female response to males, we would expect that females that mate with more attractive males would produce attractive sons with stronger immunity (as far as attractiveness and immunity are genetically based). Actually, such genetic correlation between attractiveness and viability is not required for the good genes hypothesis; a phenotypic relationship between attractiveness and offspring viability is sufficient to support the good genes hypothesis (Achorn & Rosenthal, 2020). However, the genetic-level association between overall attractiveness and viability may be a strong driver for the operation of good genes hypothesis. While some reviews have suggested that the role of indirect genetic benefits in the evolution of mate choice may have been overemphasized (Möller & Alatalo, 1999; Prokop et al., 2012), it is crucial to reevaluate the importance of indirect genetic benefits in mate choice using the IGE framework.

### Applications of IGEs in the study of sexual conflict

The IGE framework is also useful for detecting the presence of intralocus sexual conflict. Males and females of the same species share a common genome, which typically leads to positive genetic associations for traits shared between the sexes (Poissant et al., 2010). However, males and females often differ in the trait values that contribute to peak fitness, leading to sex-specific selection pressures on shared traits and the persistence of alleles with sexually antagonistic effects—beneficial for one sex but detrimental for the other (Bonduriansky & Chenoweth, 2009). Consequently, theory has suggested that fitness-related traits are likely to exhibit negative genetic correlations between males and females (Bonduriansky & Chenoweth, 2009). In line with these theoretical predictions, evidence of intralocus sexual conflict has mainly been derived from studies demonstrating negative cross-sex DGE–DGE correlations for fitness (but see Connallon & Matthews, 2019). Moreover, we propose that within-sex DGE–IGE correlations or cross-sex IGE–IGE correlations of fitness-related traits can also serve as indicators of intralocus sexual conflict (correlations d, g, and h in Figure 1). For example, intralocus sexual conflict can be identified through negative cross-sex genetic associations for overall attractiveness, as measured by IGEs on the opposite-sex response. It can also be identified by a negative genetic correlation between the overall attractiveness of the one sex (measured by IGEs on the mating behavior of the other sex) and DGEs on fitness-related traits (e.g., survival) in the other sex. In our study, we found a positive cross-sex genetic correlation between fitness-related traits (female attractiveness and male testis size). In addition, there was no association between overall attractiveness at the precopulatory stage in males and females (IGEs on female latency to mount and IGEs on male latency to sing). These findings suggest the absence of sexual conflict in *G. bimaculatus*, although this might be because of the lack of power in our study to detect IGEs with low errors (Bijma, 2010). Therefore, further investigation is needed to elucidate the relationship between sexual antagonism and cross-sex genetic correlations for fitness using the IGE framework.

## Conclusion

This study highlights that the incorporation of the IGE framework enables the assessment of overall attractiveness and testing hypotheses on the evolution of mate choice using complex within-sex or cross-sex correlations among multiple DGEs and IGEs. First, we aimed to estimate the genetic association between overall attractiveness, measured by IGEs on the response of the opposite sex, and other (sexual) traits within and between sexes. We have shown that genetic correlations between overall attractiveness (measured by IGEs on the response of the opposite sex) and potential attractive traits within a sex can reveal the specific traits shaping overall attractiveness. Additionally, the within-sex or cross-sex genetic correlation structures between overall attractiveness and other fitness-related traits can shed light into the understanding of mate choice evolution mechanisms. Therefore, we emphasize that applying a variance-partitioning approach and the IGE framework to mate choice has broad applications in addressing various evolutionary questions concerning mate choice evolution, sexual conflict, and life-history trade-offs.

## Supplementary material

Supplementary material is available online at *Evolution Letters*.

qrae037_suppl_Supplementary_Material

## Data Availability

Data and codes are available at https://doi.org/10.5061/dryad.7m0cfxq41.
