# Supplementary material for: Unraveling mate choice evolution through indirect genetic effects
Source: Evol Lett. 2024 Jul 22;8(6):841–50. doi: 10.1093/evlett/qrae037 (PMC11637604; doi:10.1093/evlett/qrae037)
Supplement: qrae037_suppl_Supplementary_Material [file qrae037_suppl_supplementary_material.pdf]

Supplementary materials

Unravelling mate choice evolution through indirect genetic effects

Chang S. Han[1*], Diana Robledo-Ruiz[2,3], Francisco Garcia-Gonzalez[4,5], Niels Dingemanse[2], Cristina Tuni[2,6]

[1] Department of Biology, Kyung Hee University, Seoul, Korea
[2] Department of Biology, Ludwig Maximilian University, Munich, Germany
[3] School of Biological Sciences, Monash University, Clayton, VIC, Australia
[4] Estación Biológica de Doñana-CSIC, Seville, Spain
[5] Centre for Evolutionary Biology, School of biological Sciences, University of Western Australia, Crawley, WA, Australia
[6] Department of Life Science & Systems Biology, University of Turin, Torino, Italy

[*] corresponding author: hcspol@gmail.com

Text S1. Maintenance of the stock population

Animals were kept in groups (of approximately 30 individuals) in plastic tanks (19 × 46 × 28.5 cm^3) equipped with egg cartons for shelter, cotton-plugged water vials and *ad libitum* dry bird food (Aleckwa Delikat, Germany) and fresh apple slices. Once they reached sexual maturity, we placed a plastic cup (diameter by height: 7 × 4.5 cm) in each tank. This cup contained moist soil for female oviposition. After one week, oviposition cups were transferred to plastic boxes (6 × 9 × 9 cm^3) for nymphs to hatch. We then randomly selected approximately 30 nymphs and transferred them to large plastic tanks, housing them in groups.

Text S2. Maintenance of sires, dams and offspring

A sire and a dam were placed together for 3 days in a tank (10 × 8 × 14 cm) equipped with shelter, food, water and an oviposition cup. The individuals were then separated, and the male was moved to the housing container of a second female (similarly equipped). Oviposition cups were kept separate, as described above, until the eggs hatched. After mating with these two females, the procedure was repeated (i.e., males were mated again with each of these two unrelated females) to ensure offspring production if a clutch from the first mating failed to hatch. The second clutch from a dam was discarded if more than 50 offspring hatched from the first clutch.

Approximately 5-6 days after hatching, 40 nymphs were isolated from each oviposition cup and divided into two plastic tanks (13 × 15 × 22 cm) containing 20 nymphs each. Nymphs were provided with shelter, food and water and reared in full-family groups until the penultimate instar. Subsequently, male offspring were housed individually (as above) to control mating and social experience until sexual maturation; females were kept in family tanks (approximately 10 individuals per tank) of same-age individuals (i.e., females moulting on the same day were relocated to the same tank).

Text S3. Dissections and measurements of morphological traits

To measure morphological traits, male and female crickets were thawed at room temperature for 20 minutes. The specimens were then placed under a stereomicroscope (Zeiss), with the head pinned, and the mandibles carefully excised using microscissors. The mandibles were positioned on 1-mm^2 grid paper and photographed using a tablet camera (MOTICON) connected to a microscope (Zeiss) at a magnification of 0.65. The procedure outlined in Judge and Bonanno (2008) was followed, and ImageJ software was employed for precise measurement of mandible size to the nearest 0.01 mm from the images. Subsequently, the abdomen was dissected, and the testes were extracted using forceps. Testes weight was determined using a microbalance (Sartorius) to the nearest 0.001 g.

Text S4. Total heritable variance

Methods The total heritable variance (V_{TBV}) includes all the heritable variance on which selection can act, incorporating heritable variance present in the social environment. For traits affected by interactions among individuals, the total heritable variance includes heritable social effects such as IGEs and the DGE-IGE covariance as well as DGEs (Bijma et al. 2007). If the DGE-IGE covariance and IGE are positive, the total heritable variance is greater than classical heritable variance (DGEs). In our study, we estimated the total heritable variance using the equation ($V_{TBV} = V_{Am} + V_{Af} + 2COV_{Am,Af}$) which specifically holds for a dyadic interaction (group number(n) = 2; equation (6) in (Bijma et al. 2007)).

Results and Discussion As IGEs can be additional additive genetic components of socially interacting phenotypes, the amount of additive genetic variance in these traits can be greater than that from DGEs alone, potentially accelerating their evolution (Bijma et al. 2007; Bijma and Wade 2008; Bijma 2011; Bijma 2014; Wilson 2014). In our study, despite the non-significant covariance between DGEs and IGEs for male latency to sing, both DGEs and IGEs were significant. This resulted in a greater total heritable variance in male latency to sing (V_{TBV} (SE) = 0.22 (0.12)) compared to the variance from DGEs alone (V_{Am} (SE) = 0.12 (0.04), Figure S1). Similarly, although DGEs for female latency to mount were not significant (V_{Af} (SE) = 0.06 (0.05), Tables S1), significant IGEs contributed to a positive total heritable variance in female latency to mount (V_{TBV} (SE) = 0.14 (0.07), Figure S1). Consequently, even in the absence of significant DGEs on female latency to mount, IGEs allow this trait to respond to selection.

Our results highlight the importance of considering IGEs in predicting the evolution of socially interacting phenotypes, such as mating behaviour. Moreover, IGEs, like DGEs, can be associated with additive genetic components of other phenotypes within or across sexes, potentially making the evolution of socially interacting phenotypes more complex (see the main text).

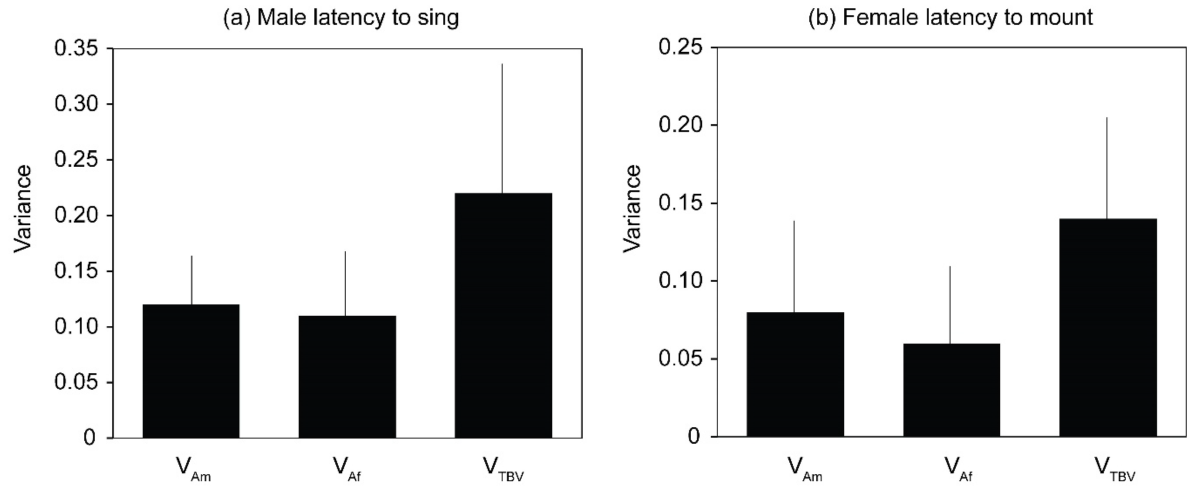

Figure S1. Variance components (V_{Am}, additive genetic effect of males; V_{Af}, additive genetic effect of females) and total heritable variance ($V_{TBV} = V_{Am} + V_{Af} + 2COV_{Am,Af}$, Equation (6) in (Bijma et al. 2007)) of (a) male latency to sing and (b) female latency to mount. The mean variance estimates are provided with the associated standard errors (error bars).

Table S1. Linear mixed model of behavioural and morphological traits. Parameters are provided with standard errors (SEs) in parentheses. V_{Am}, additive genetic effect of males; V_{PEm}, permanent environmental effect of males; V_{Af}, additive genetic effect of females; V_{PEf}, permanent environmental effect of females; $COV_{Am,Af}$, covariance between additive genetic effects of males and females; V_R, residual variance.

	**Male latency to sing**			**Female latency to mount**			**Male guarding intensity**		
Fixed effects	β (SE)	$F_{NUMdf, DENdf}$	P	β (SE)	$F_{NUMdf, DENdf}$	P	β (SE)	$F_{NUMdf, DENdf}$	P
**Intercept**	0.07 (0.15)	$0.33_{1,342.8}$	0.57	-0.06 (0.12)	$0.79_{1,261.2}$	0.37	0.19 (0.16)	$0.01_{1,303.0}$	0.93
**Testing order**	-0.03 (0.05)	$0.40_{1,467.0}$	0.53	0.05 (0.05)	$1.14_{1,362.7}$	0.29	-0.01 (0.06)	$0.06_{1,352.7}$	0.81
**Shelf**	0.02 (0.08)	$0.06_{1,598.4}$	0.81	-0.07 (0.08)	$0.71_{1,579.2}$	0.40	-0.28 (0.09)	$10.55_{1,512.4}$	0.001
Random effects	σ^2 (SE)	$\chi^2_{0/1}$‡	P	σ^2 (SE)	$\chi^2_{0/1}$‡	P	σ^2 (SE)	$\chi^2_{0/1}$‡	P
$\mathbf{V_{Am}}$	0.12 (0.04) [a]	11.39	<0.001	0.08 (0.06) [c]	2.89	0.04	0.00 (-) [e]	0.00	0.50
$\mathbf{V_{PEm}}$	0.00 (-)	0.00	0.50	0.06 (0.07)	0.70	0.20	0.09 (0.06)	2.51	0.06
$\mathbf{V_{Af}}$	0.10 (0.06) [b]	3.28	0.03	0.06 (0.05) [d]	1.58	0.10	0.08 (0.07) [f]	1.57	0.11
$\mathbf{V_{PEf}}$	0.32 (0.14)	2.51	0.06	0.00 (-)	0.00	0.50	0.27 (0.15)	2.69	0.05
$\mathbf{COV_{Am, Af}}$	0.00 (0.04)	0.00	0.50						
$\mathbf{V_R}$	0.48 (0.12)			0.80 (0.08)			0.56 (0.14)		

	**Male body mass**			**Male testis size**			**Male mandible size**			**Female body mass**		
Fixed effects	β (SE)	$F_{NUMdf, DENdf}$	P	β (SE)	$F_{NUMdf, DENdf}$	P	β (SE)	$F_{NUMdf, DENdf}$	P	β (SE)	$F_{NUMdf, DENdf}$	P
**Intercept**	0.13 (0.10)	$1.67_{1,51.4}$	0.20	0.03 (0.09)	$0.09_{1,42.7}$	0.76	0.02 (0.09)	$0.07_{1,41.3}$	0.80	0.11 (0.10)	$1.18_{1,640.1}$	0.28
**Testing order**	-0.03 (0.01)	$5.73_{1,514.1}$	0.02							-0.01 (0.04)	$0.07_{1,818.7}$	0.80
Random effects	σ^2 (SE)	$\chi^2_{0/1}$‡	P	σ^2 (SE)	$\chi^2_{0/1}$‡	P	σ^2 (SE)	$\chi^2_{0/1}$‡	P	σ^2(SE)	$\chi^2_{0/1}$‡	P
$\mathbf{V_{Ad}}$	0.69 (0.19)	47.54	<0.001	0.53 (0.17)	25.16	<0.001	0.47 (0.16)	20.04	<0.001	0.39 (0.08)	85.46	<0.001
$\mathbf{V_{PEd}}$	0.29 (0.12)	4.39	0.02							0.00 (0.00)	0.00	0.50
$\mathbf{V_R}$	0.06 (0.01)			0.49 (0.12)			0.55 (0.12)			0.62 (0.05)		

[a] DGE on male latency to sing
[b] IGE on male latency to sing
[c] IGE on female latency to mount
[d] DGE on female latency to mount
[e] DGE on male guarding intensity
[f] IGE on male guarding intensity

Table S2. Additive genetic (co)variance and correlation matrix. Narrow-sense heritability is displayed on the diagonal, genetic correlations are displayed on the upper off-diagonal and additive genetic covariances are displayed on the lower off-diagonal. Values in parentheses are standard errors of additive genetic (co)variances and correlations. Latency values were reverse scored, as indicated by an asterisk (*).

			Male				Female			
			Latency to sing *	Latency to sing *	Body mass	Testis size	Mandible size	latency to mount *	latency to mount *	Body mass
			DGE	IGE [a]	DGE	DGE	DGE	DGE	IGE [b]	DGE
Male	Latency to sing *	DGE	0.12 (0.04)	0.02 (0.40)	0.01 (0.16)	-0.19 (0.20)	0.06 (0.19)	-	0.99 (-)	0.05 (0.26)
	Latency to sing *	IGE [a]	0.00 (0.04)	0.11 (0.06)	0.03 (0.08)	0.15 (0.07)	0.06 (0.07)	-	0.05 (0.04)	-0.09 (0.20)
	Body mass	DGE	0.00 (0.04)	0.11 (0.31)	0.70 (0.19)	0.44 (0.10)	0.95 (0.03)	-	0.03 (0.05)	0.53 (0.16)
	Testis size	DGE	-0.04 (0.05)	0.67 (0.30)	0.29 (0.07)	0.53 (0.17)	0.47 (0.11)	-	-0.05 (0.05)	0.21 (0.20)
	Mandible size	DGE	0.01 (0.05)	0.26 (0.33)	0.94 (0.09)	0.30 (0.08)	0.47 (0.16)	-	0.03 (0.05)	0.56 (0.18)
Female	Latency to mount *	DGE	-	-	-	-	-	0.06 (0.05)	-	-
	Latency to mount *	IGE [b]	0.31 (0.05)	0.56 (0.48)	0.11 (0.19)	-0.27 (0.23)	0.15 (0.22)	-	0.08 (0.06)	-0.05 (0.30)
	Body mass	DGE	0.01 (0.06)	-0.02 (0.04)	0.27 (0.10)	0.10 (0.10)	0.24 (0.10)	-	-0.01 (0.06)	0.39 (0.08)

[a] Indicates (1) genetic variation in female attractiveness and (2) the existence of male preference for specific female genotypes.
[b] Indicates (1) genetic variation in male attractiveness and (2) the existence of female preference for specific male genotypes.

References

Bijma, P. 2011. A general definition of the heritable variation that determines the potential of a population to respond to selection. Genetics 189:1347-1359.
Bijma, P. 2014. The quantitative genetics of indirect genetic effects: a selective review of modelling issues. Heredity 112:61-69.
Bijma, P., W. M. Muir, and J. A. Van Arendonk. 2007. Multilevel selection 1: quantitative genetics of inheritance and response to selection. Genetics 175:277-288.
Bijma, P. and M. Wade. 2008. The joint effects of kin, multilevel selection and indirect genetic effects on response to genetic selection. J. Evol. Biol. 21:1175-1188.
Wilson, A. 2014. Competition as a source of constraint on life history evolution in natural populations. Heredity 112:70-78.
